# Activation of the microglial P2X7R/NLRP3 inflammasome mediates central sensitization in a mouse model of medication overuse headache

**DOI:** 10.3389/fnmol.2023.1177171

**Published:** 2023-06-12

**Authors:** Yanyun Wang, Liang Dong, Yun Zhang, Yixin Zhang, Guangcheng Qin, Dunke Zhang, Lixue Chen, Wei He, Jiying Zhou

**Affiliations:** ^1^Department of Neurology, The First Affiliated Hospital of Chongqing Medical University, Chongqing, China; ^2^Laboratory Research Center, The First Affiliated Hospital of Chongqing Medical University, Chongqing, China; ^3^Department of Neurology, The First Branch of The First Affiliated Hospital of Chongqing Medical University, Chongqing, China

**Keywords:** microglia, medication overuse headache, central sensitization, P2X7R, NLRP3 inflammasome

## Abstract

**Background:**

Excessive use of headache treatments often leads to the development, progression and exacerbation of primary headache, which is defined as medication overuse headache (MOH). A significant pathophysiological mechanism of MOH is central sensitization. Recent evidence suggests that central sensitization in chronic headache is a result of inflammatory responses mediated by microglial activation in the trigeminal nucleus caudalis (TNC). However, it is unknown whether microglial activation has an impact on the central sensitization of MOH. Accordingly, the goal of this research was to determine how microglial activation and the P2X7R/NLRP3 inflammasome signaling pathway in the TNC contribute to the pathogenesis of MOH.

**Methods:**

Repeated intraperitoneal injection of sumatriptan (SUMA) was used to establish a mouse model of MOH. Basal mechanical hyperalgesia was evaluated using von Frey filaments. As central sensitization biomarkers, the c-Fos and CGRP expression levels were measured by immunofluorescence analysis. We estimated the expression of microglial biomarkers (Iba1 and iNOS) within the TNC by qRT-PCR, western blotting and immunofluorescence analysis. To elucidate the effect of microglial activation and the P2X7/NLRP3 signaling pathway on central sensitization in MOH, we evaluated whether the microglia-specific inhibitor minocycline, the P2X7R-specific antagonist BBG and the NLRP3-specific inhibitor MCC950 altered SUMA-caused mechanical hyperalgesia. Furthermore, we examined c-Fos and CGRP expression within the TNC following individual injections of these inhibitors.

**Results:**

Repeated SUMA injection induced basal mechanical hyperalgesia, increased c-Fos and CGRP levels, and activated microglia within the TNC. Inhibiting microglial activation with minocycline prevented the emergence of mechanical hyperalgesia and cut down c-Fos and CGRP expression. Immunofluorescence colocalization analysis revealed that P2X7R was predominantly co-localized with microglia. The levels of P2X7R and the NLRP3 inflammasome were elevated by repeated SUMA injection, and blocking P2X7R and NLRP3 inhibited mechanical hyperalgesia and cut down c-Fos and CGRP expression within the TNC.

**Conclusion:**

Based on the current findings, inhibiting microglial activation could reduce central sensitization caused by chronic SUMA treatment *via* the P2X7R/NLRP3 signaling pathway. The clinical management of MOH may benefit from a novel strategy that inhibits microglial activation.

## 1. Introduction

Migraine, as one of the most widespread headache disorders, affects approximately 1 billion individuals worldwide and 2.5% of cases become chronic from episodic migraine each year (Bigal et al., 2008). One of the primary contributors for the chronicity of migraine has been demonstrated to be frequent use of strong painkillers, which has also led to the emergence of a novel type of secondary headache termed as medication overuse headache (MOH) (Ferrari et al., 2015). MOH is defined as the frequent monthly use of acute analgesics (for more than 3 months) on top of the primary headache, which causes the exacerbation of the existing headache or the emergence of a new form of headache, in accordance with the International Classification of Headache Disorders, Third Edition (ICHD-3) (Olesen, 2018; Russell, 2019). The prevalence of MOH is approximately 1–2%, and it is characterized by severe symptoms, has a high recurrence rate, and is difficult to treat, placing a huge burden on individuals and society (Collaborators, 2018; Diener et al., 2019; Russell, 2019).

In the ICHD-3, the diagnosis of MOH is subdivided according to the drugs used, including simple analgesics such as acetaminophen or NSAIDs for at least 15 days each month and triptans, ergot derivatives or mixed analgesics for at least 10 days each month. Although there are several medications that can cause MOH, triptans are more commonly used to treat migraine patients in clinical practice (Oh et al., 2022). Patients who overuse triptans develop MOH more quickly than those who use other drugs, such as ergotamines, and have headaches that are more similar to migraine (Limmroth et al., 2002). Similarly, in early animal studies of MOH, mechanical pain threshold reduction was observed in mice that were repeatedly exposed to sumatriptan (SUMA) (de Felice et al., 2010b).

To date, the pathophysiological mechanisms of MOH remain unknown. Animal studies have shown that medication overuse makes neurons in the trigeminal pathway more excitable, resulting in central sensitization. The expression of neuropeptides was found to be increased within the trigeminal vascular system in rats that were overexposed to SUMA (de Felice et al., 2010b). According to recent research, the mechanism of central sensitization in the central nervous system (CNS) is associated not only with neurons but also with microglia (Magni et al., 2020). Activated microglia release a multitude of inflammatory factors that can interact with neurons or microglia to form a positive feedback loop (Johnson et al., 2013). Chronic morphine exposure has been demonstrated to activate spinal microglia and that activated microglia lead to morphine analgesic tolerance by activating downstream inflammatory factors (Zhou et al., 2010).

P2X7 receptor (P2X7R), an ionic receptor belonging to the purinergic P2 receptor family, is prominently generated in microglia. The activation of microglial P2X7R has been shown to have an effect on a number of pain-related conditions, including neuropathic pain, cancer pain, and inflammatory pain (Liu et al., 2015; Yang et al., 2015; Hu et al., 2020). The NLRP3 inflammasome is an inborn immunomodulatory compound consisting of nucleotide-binding oligomeric domain-like receptor protein 3 (NLRP3), cysteine-aspartate protease-1 (Caspase-1) and apoptosis-associated speck-like protein containing CARD (ASC). P2X7R can act on the NLRP3 inflammasome and contribute to central sensitization by inducing the NLRP3 inflammasome recruitment and activation to mediate IL-1β maturation (Franceschini et al., 2015; Pelegrin, 2021). Our previous studies verified that microglial P2X7R and the NLRP3 inflammasome in the trigeminovascular system participate in the central sensitization of chronic migraine induced by nitroglycerin (NTG) (He et al., 2019; Jiang et al., 2021).

Therefore, we emphasized on examining the function of microglia in a mouse model of MOH in the present study. We found that microglia were activated in the trigeminal nucleus caudalis (TNC) after repeated treatment of SUMA and that inhibiting microglial activation attenuated mechanical hyperalgesia and central sensitization in MOH mice. To further examine the underlying molecular mechanisms, we investigated the involvement of microglial P2X7R and the NLRP3 inflammasome. Our results demonstrated that activated P2X7R promoted central sensitization and mechanical hyperalgesia in MOH by modifying the NLRP3 inflammasome.

## 2. Materials and methods

### 2.1. Animals

The Animal Care and Use Committee of Chongqing Medical University in China gave its approval to all the experiments of this study, which were carried out in line with the NIH Guide for the Care and Use of Laboratory Animals. We used female and male C57BL/6 mice weighed 18–25 g and aged 6–8 weeks old. The mice were kept under a typical laboratory setting with alternating 12/12-h light/dark cycles at 22 ± 2°C and 50 ± 5% relative humidity. Food and water were easily accessible at any time. All experiments were conducted in a blinded manner. Before conducting any experimental procedures, the animals were given at least a week to acclimate to their surroundings and were randomly divided into different testing groups. All mice were kept in groups (6 per cage) to avoid the stress of social isolation.

### 2.2. MOH mouse model and drug administration

**Figure 1:**
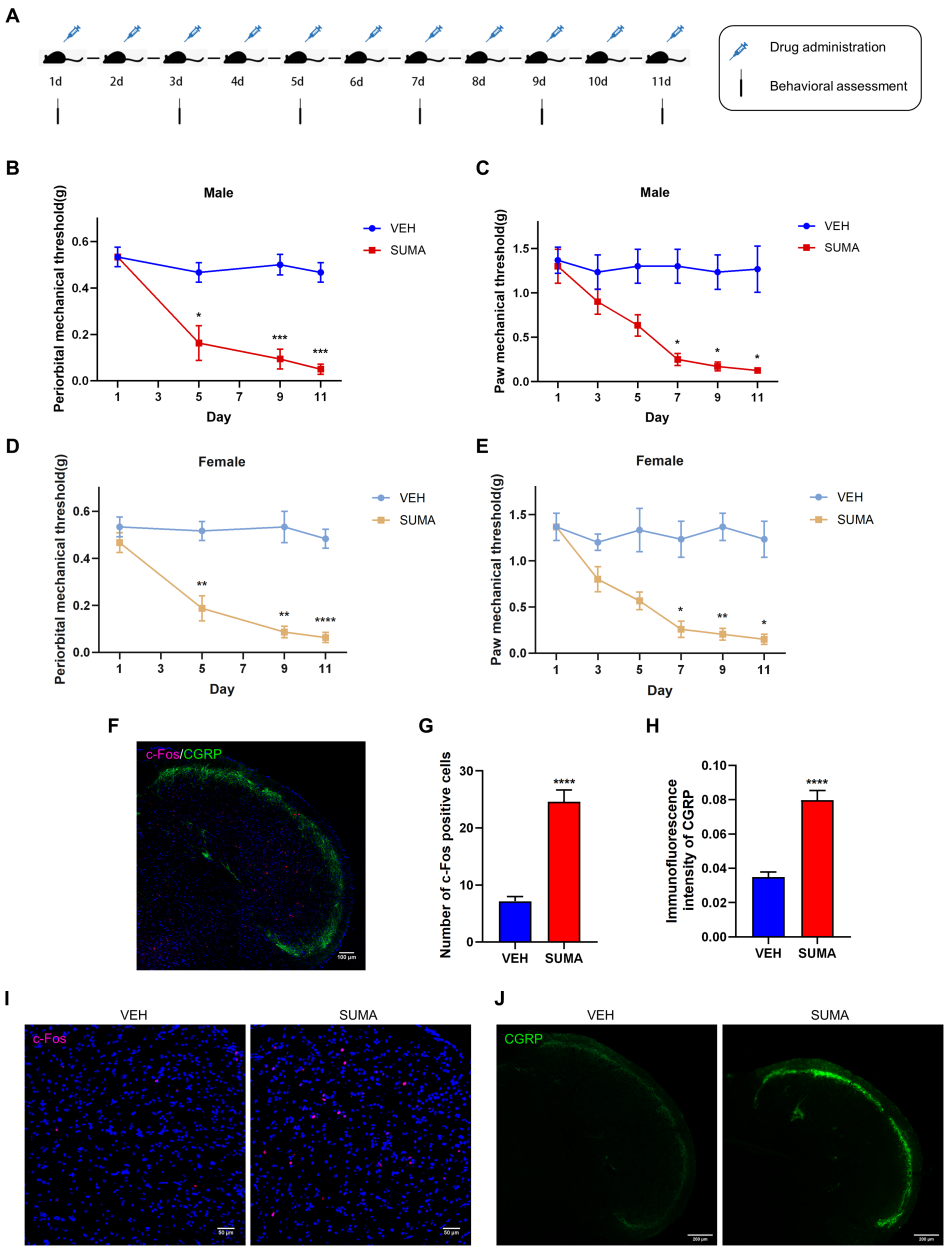
Repeated SUMA injection caused hyperalgesia and upregulated c-Fos and CGRP expression in the TNC. **(A)** Schematic timeline of the drug injections and behavioral assessments. **(B,C)** In male mice, the periorbital and hind paws mechanical pain thresholds were significantly reduced in the SUMA group compared with the VEH group, *n* = 6 per group. **(D,E)** In female mice, the periorbital and hind paws mechanical pain thresholds were also considerably decreased in the SUMA group compared with the VEH group, *n* = 6 per group. **(F)** Image of double immunofluorescence labeling of c-Fos and CGRP in TNC. **(G,I)** c-Fos immunofluorescence staining of different groups. The quantity of c-Fos- immunopositive cells was relatively larger in the SUMA group than in the VEH group, *n* = 4 per group. **(H,J)** CGRP immunofluorescence staining of different groups. The CGRP fluorescence intensity was substantially higher in the SUMA group than in the VEH group, *n* = 4 per group (*p < 0.05, **p < 0.01, ***p < 0.001 and ****p < 0.0001, * represents that the SUMA group was compared with the VEH group; scale bar = 50/100/200 μm).

A MOH model induced by reiterant SUMA injection was developed based on previous studies (Moye et al., 2019; Saengjaroentham et al., 2020; Zhang J. et al., 2020). Prior to being injected, sumatriptan succinate (MedChemExpress/MCE, USA) was diluted in 0.9% saline. Using 0.9% saline as a vehicle control, animals were administered 0.6 mg/kg SUMA or the equivalent vehicle by intraperitoneal injection (i.p.) once daily for 11 days (Figure 1A).

The microglial inhibitor minocycline (MedChemExpress/MCE, United States) was used to study the role of microglia in MOH. Similar to SUMA, minocycline was administered intraperitoneally after being dissolved in sterile saline at a daily quota of 30 mg/kg for 11 days. The function of P2X7R in MOH was investigated using Brilliant Blue G, a P2X7R specific inhibitor (Sigma Aldrich, Hungary). BBG was injected intraperitoneally at a dosage of 50 mg/kg daily for 11 days after being diluted in 0.9% saline. The NLRP3 inhibitor MCC950 (MedChemExpress/MCE, USA) was used to study the function of NLRP3 in MOH. MCC950 was soluble in 0.9% saline and given to mice at a dosage of 10 mg/kg daily for 11 days. As a vehicle control, an identical volume of solvent was used. The literature and early experiments were used to determine the drug dosage and injection method. On the day of use, all chemical solutions were freshly prepared.

### 2.3. Behavioral tests

From 9:00 to 15:00, all behavioral assessments were conducted in a calm, dimly lit atmosphere. In the present study, behavioral assessments included periorbital and hind paw mechanical withdrawal thresholds, which also served as markers of central sensitization (Bonnet et al., 2019; Tian et al., 2022). All behavioral tests were performed on the same group of animals. All mice were received training for 3 days prior to the experiment and were tested before injection to establish a reliable baseline response. The mechanical withdrawal threshold was measured by the up-and-down technique with a graded von Frey filament (0.02–2 g) applied to the periorbital or hind paw region. Testing was performed on the hind paw every second day after injections were initiated (Figure 1A). For the periorbital area, testing was performed every 1–3 days after injections were initiated, as excessive facial irritation may cause allergic reactions. The evaluators had no knowledge of the experimental groupings, and there were six mice per group.

### 2.4. Quantitative real-time polymerase chain reaction (qRT-PCR)

As mentioned in earlier research, qRT-PCR was carried out (Zhang Y. et al., 2020). The TNC tissues were swiftly removed and temporarily kept in liquid nitrogen after the mice were given 1% sodium pentobarbital to render them completely comatose. RNAiso Plus reagent (TaKaRa, Dalian, China) was employed to purify total RNA, and a NanoDrop spectrophotometer (Thermo, United States) was utilized to quantify the OD260. Using the PrimeScriptTM RT reagent kits (TaKaRa, Dalian, China), cDNA was created by reverse transcription. Quantitative polymerase chain reaction was conducted on a CFX96 Touch thermal cycler (Bio-Rad) using a SYBR Premix Ex Taq II kit (TaKaRa, Dalian, China). A post-PCR data processing program was used to analyze all fluorescence data, and the 2-ΔΔCT method was applied to calculate the relative change of gene expression. There were 5–6 mice in each group. In Table 1, the primer sequences are presented. Glyceraldehyde-3-phosphate dehydrogenase (GAPDH) was utilized as a control.

**Table 1:** Primer sequences in qRT-PCRs.

Gene	Forward primer (5′ → 3′)	Reverse primer (5′ → 3′)
iNOS	ATCTTGGAGCGAGTTGTGGATTGTC	TAGGTGAGGGCTTGGCTGAGT
P2X7R	CAGCGGAAAGAGCCTGTTATC	TGGCCTTCTGACTTGACATAGTT
NLRP3	GCCGTCTACGTCTTCTTCCTTTCC	CATCCGCAGCCAGTGAACAGAG
GAPDH	GGTTGTCTCCTGCGACTTCA	TGGTCCAGGGTTTCTTACTCC

### 2.5. Western blot analysis

The TNC tissues were promptly removed and kept at −80°C after the mice were given 1% sodium pentobarbital to give them a deep anesthesia. The tissue was homogenized for 1 h at 4°C after the addition of radioimmunoprecipitation assay buffer (Beyotime, Shanghai, China) including a protease inhibitor and phenylmethylsulfonyl fluoride (PMSF). The protein concentrations were detected by BCA Protein Assay Kits (Beyotime, Shanghai, China). Equal amounts of protein (20–40 g) were electrotransferred to PVDF membranes (Millipore, United States) after being segregated on 10% or 12% SDS-PAGE gels (Beyotime, Shanghai, China). After being blocked with 5% skimmed milk at room temperature for 2 hours, the membranes were first mixed with primary antibodies (Table 2) for an overnight period at 4°C. The following day, after being rinsed in TBST, the membranes were coated with secondary antibodies (goat-anti-rabbit and goat-anti-mouse, ZSGBIO, China) for 1 hour at room temperature. With the aid of ECL assay kits (Advansta Inc., United States) and an imaging system (Fusion, Germany), immunoreactive bands were visualized and analyzed. There were six mice in each group.

**Table 2:** Antibodies used for western blotting and immunofluorescence staining.

Antibody	Manufacturer	Catalog number	Host	Dilution
For western blot analysis				
P2X7	Alomone, Israel	APR-004	Rabbit	1:1000
NLRP3	Abcam, UK	ab263899	Rabbit	1:1000
Caspase-1	Proteintech, China	22,915-1-AP	Rabbit	1:500
IL-1β	Bioss, China	bs-0812R	Rabbit	1:2000
Iba1	Abclonal, China	A1527	Rabbit	1:1000
iNOS	Proteintech, China	18,985-1-AP	Rabbit	1:1500
GAPDH	ZEN-BIOSCIENCE, China	200,306-7E4	Mouse	1:5000
For immunofluorescence staining				
P2X7	Alomone, Israel	APR-004	Rabbit	1:200
CGRP	Santa Cruz, USA	sc-57,053	Mouse	1:100
c-Fos	Novus Biologicals, USA	NBP2-50057SS	Rabbit	1:5000
Iba1	Abcam, UK	ab178846	Rabbit	1:200
Iba1	Thermo, USA	MA5-27726	Mouse	1:200
NeuN	Proteintech, China	66,836-1-Ig	Mouse	1:100
GFAP	Santa Cruz, USA	sc-33,673	Mouse	1:200

### 2.6. Immunofluorescence staining

The animals were given 1% sodium pentobarbital to induce a profound anesthesia before receiving 60 mL of precooled phosphate-buffered saline (PBS) and 60 mL of precooled 4% paraformaldehyde transcardially (PFA). The cervical spinal cord and brainstem were taken, covered with 4% PFA, and left to fix at 4°C for 24 h. The tissue was then further dehydrated in sucrose solutions of 20 and 30% until it sank. After being embedded, the tissue was quickly frozen. In accordance with the mouse brain atlas (Pinskiy et al., 2013), TNC tissues were divided into 10–25 μm sections. The slices were repaired with sodium citrate, infiltrated for 10 min with 0.3% Triton X-100 (Beyotime, Shanghai, China), and then sealed for 30 min with 5% goat serum (Boston, Wuhan, China) at 37°C. After that, the slices were reacted with primary antibodies (Table 2) overnight at 4°C. The next day, after being rinsed in PBS, the slices were coated with the appropriate secondary antibodies (Alexa Fluor cy3 or 488) at 37°C for 60 min. The slices were treated with a combination of primary or secondary antibodies from two distinct sources for double immunofluorescence analysis. 4′,6-diamidino-2-phenylindole (DAPI) was utilized to stain the nuclei at 37°C for 10 min. A confocal microscope (LSM800, Zeiss, Germany) was employed to capture images. All images were taken under uniform parameters (100 × objective), and the fluorescence signal intensity was quantified using image analysis software (Image J 1.8.0). The fluorescence intensity of CGRP was calculated using the mean optical density (OD) of CGRP (Zhang Y. et al., 2020). The quantity of Iba1, c-fos immunopositive cells was also counted by ImageJ. Four mice each group, with 4–5 slices per mouse, were assessed. An experimenter who was not aware of the experimental groupings collected and analyzed the images.

### 2.7. Statistical analysis

For statistical analysis and graph creation, GraphPad Prism version 8.0 (GraphPad Software Inc., San Diego, CA, United States) was applied. The Kolmogorov–Smirnov test was employed to determine the data’s normality before statistical analysis. All data are expressed as the mean ± standard error (SEM). In addition to behavioral data, data from two groups were compared using Student’s t tests. One-way analysis of variance (ANOVA) and Tukey or Dunnett *post hoc* tests were utilized for multiple comparisons. For the examination of behavioral data, two-way ANOVA and Bonferroni *post hoc* tests were employed. Statistics were judged significant when *p* < 0.05.

## 3. Results

### 3.1. Repeated SUMA injection caused hyperalgesia and upregulates c-Fos and CGRP expression in the TNC

The female and male mice were intraperitoneally injected with SUMA for 11 consecutive days to establish the MOH mouse model (SUMA group), and the control group (VEH group) was intraperitoneally injected with sterile saline for 11 consecutive days (Figure 1A). Repeated injections of SUMA over the course of 11 days in both male and female mice induced a gradual decline in periorbital and hind paws withdrawal thresholds (*p* < 0.05, Figures 1B–E). However, the VEH group did not exhibit any appreciable variation. To avoid sex differences, we used only male mice in subsequent studies to avoid possible hormonal effects. c-Fos and CGRP were chosen as the central sensitization indicators based on prior research (Latremoliere and Woolf, 2009; Iyengar et al., 2017). Double immunofluorescence labeling of c-Fos and CGRP in the TNC is shown in Figure 1F. Immunofluorescence staining revealed that the SUMA group had higher levels of c-Fos and CGRP expression than the VEH group did in the TNC (*p* < 0.0001; Figures 1G–J). Consistent with earlier research, these results demonstrated that repeated SUMA injection causes central sensitization in mice.

### 3.2. Repeated SUMA injection induced the activation of microglia in the TNC

**Figure 2:**
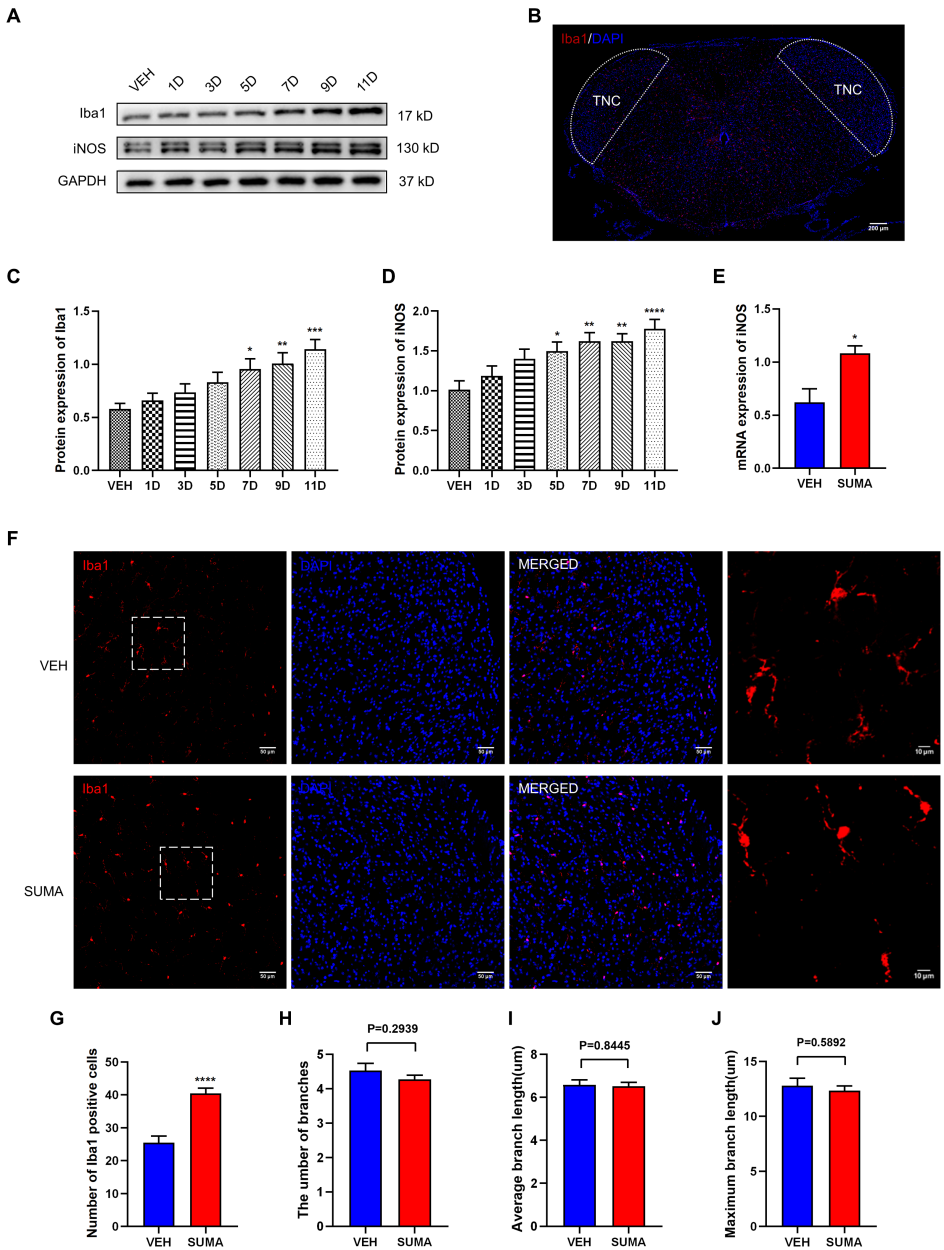
Repeated SUMA injection induced microglial activation in the TNC. **(A,C,D)** The protein expression of Iba1 and iNOS grew gradually with SUMA injection, *n* = 6 per group. **(B)** The observed TNC area is shown as the dashed line. **(E)** In comparison with the VEH group, the SUMA group had higher mRNA levels of iNOS, *n* = 6 per group. **(F)** The immunofluorescence labeling image of Iba1 in the TNC. The rightmost column of Panel **(F)** shows an enlarged view of the dashed box on the left, *n* = 4 per group. **(G)** The quantity of Iba1 immunopositive cells in the SUMA group was larger than that in the VEH group, *n* = 4 per group. **(H–J)** The number of branches, average branch length and maximum branch length of microglia (VEH vs. SUMA: 4.535 vs. 4.273, *p* = 0.2939; 6.577 um vs. 6.519 um, *p* = 0.8445, 12.79 um vs. 12.35 um, *p* = 0.5892 respectively), *n* = 4 per group (*p < 0.05, **p < 0.01, ***p < 0.001 and ****p < 0.0001, * represents that the SUMA group was compared with the VEH group; scale bar = 10/50/200 μm).

To observe changes in the activation of microglia in the TNC after repeated SUMA injection, we assessed the microglial marker Iba1 and the M1 (proinflammatory) microglial marker iNOS (Wang et al., 2021; Armeli et al., 2022). We analyzed the protein expression of Iba1 and iNOS by western blotting. Compared with that in the VEH group, the protein expression of Iba1 and iNOS in the SUMA group steadily raised with repeated injections of SUMA (*p* < 0.05; Figures 2A,C,D). Consistent with the western blot results, elevated mRNA levels of iNOS were observed in the SUMA group, as determined by qRT–PCR (*p* < 0.05; Figure 2E). The observed TNC area is shown as the dashed line in Figure 2B. Immunofluorescence staining revealed a spike in the number of Iba1 immunopositive cells after repeated SUMA injection (*p* < 0.0001, Figures 2F,G) and a shift from the resting (dendritic) to the activated state in microglia in the SUMA group as indicated by fewer protrusions (Figure 2F, rightmost column). The number of branches, average branch length and maximum branch length of microglia were analyzed, but there was no significant difference between the VEH and SUMA groups (*p* = 0.2939, *p* = 0.8445, *p* = 0.5892 respectively, Figures 2H–J) (Young and Morrison, 2018). According to these results, repeated SUMA injection activated microglia in the TNC.

### 3.3. Inhibiting microglial activation restored mechanical hyperalgesia and reduced c-Fos and CGRP expression induced by repeated SUMA injection

**Figure 3:**
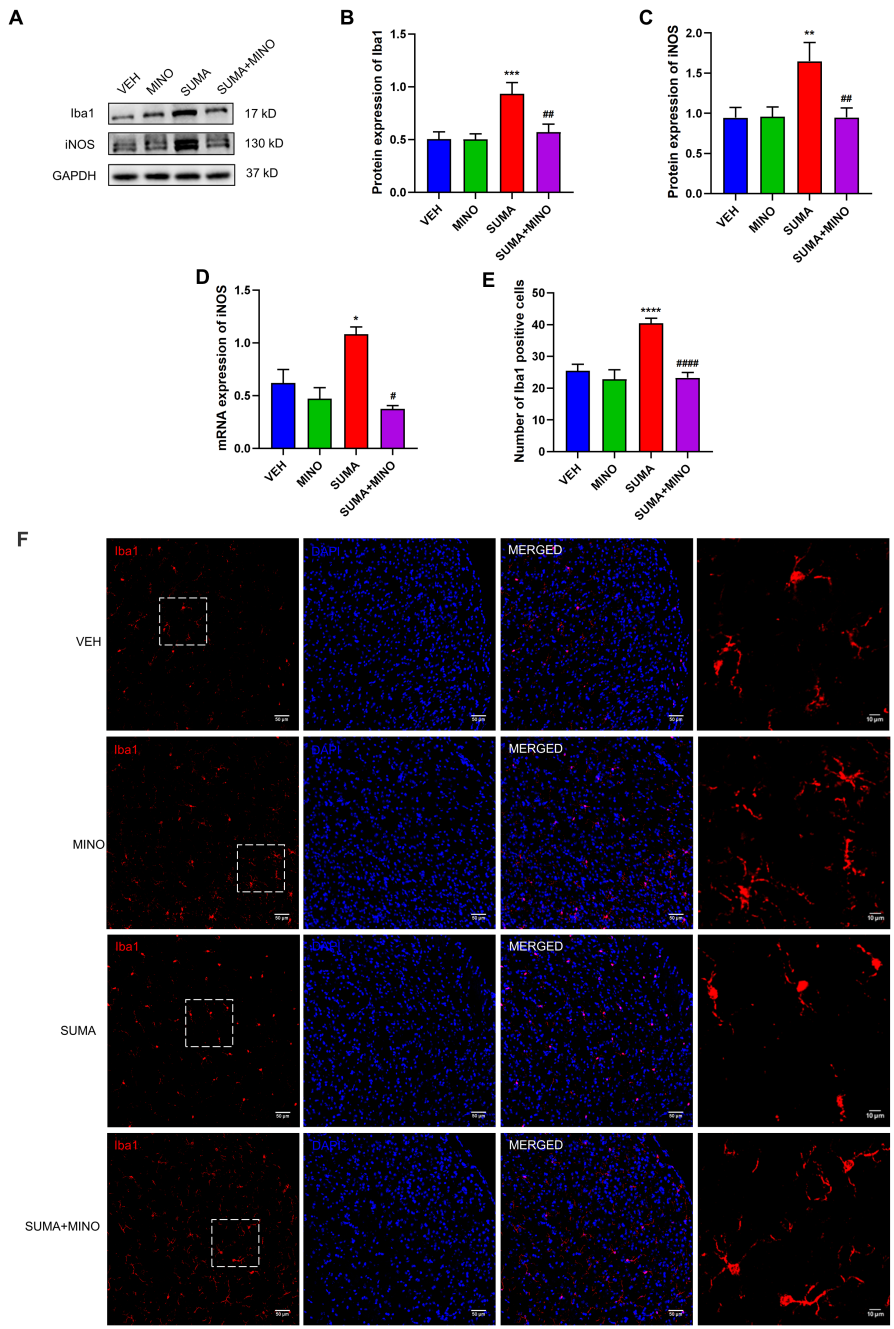
Inhibiting microglial activation with the microglial inhibitor minocycline. **(A–C)** The protein expression of Iba1 and iNOS, *n* = 6 per group. **(D)** The mRNA expression of iNOS, *n* = 6 per group. **(E,F)** Immunofluorescence labeling pictures of Iba1 in the TNC showed a significant decrease in Iba1 immunopositive cells in the SUMA+MINO group compared with the SUMA group. The rightmost column of Panel **(F)** shows an enlarged view of the dashed box on the left. The number of branches, average branch length and maximum branch length of microglia did not differ significantly between groups (SUMA vs. SUMA+MINO: 4.273 vs. 4.481, *p* = 0.5120; 6.519 um vs. 6.553 um, *p* = 0.9988; 12.35 um vs. 12.78 um, *p* = 0.8447 respectively), *n* = 4 per group (*p < 0.05, **p < 0.01, ***p < 0.001, and ****p < 0.0001; #*p* < 0.05, ^##^*p* < 0.01 and ^####^*p* < 0.0001; *represents that the SUMA group was compared with the VEH group, ^#^represents that the SUMA group was compared with the SUMA+MINO group; scale bar = 10/50 μm).

**Figure 4:**
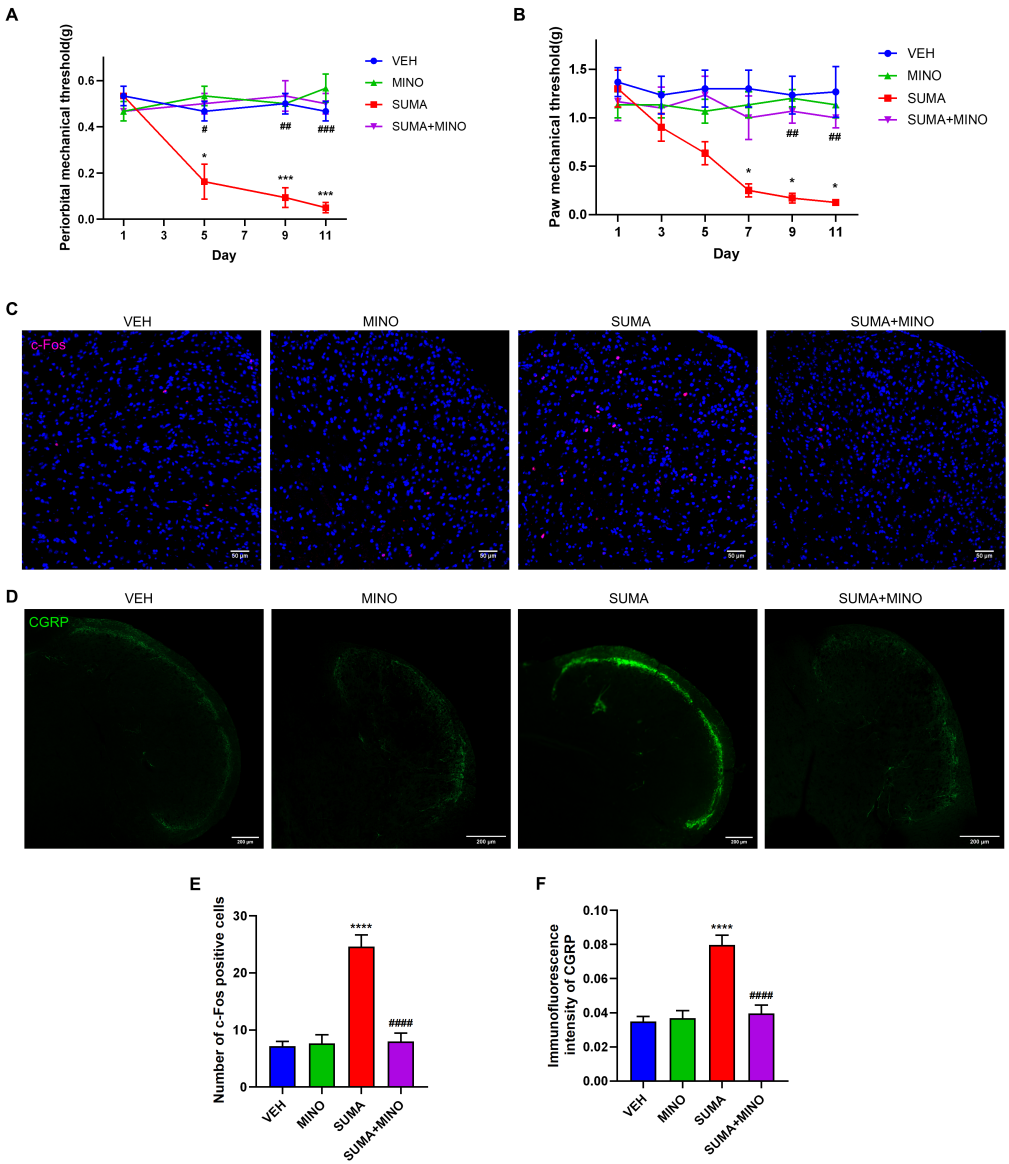
Inhibiting microglial activation restored mechanical hyperalgesia and reduced c-Fos and CGRP expression induced by repeated SUMA injection. **(A,B)** The periorbital and hind paws mechanical withdrawal thresholds were dramatically restored in the SUMA+MINO group compared with the SUMA group, *n* = 6 per group. **(C,E)** Immunofluorescence labeling of c-Fos showed a decrease in the quantity of c-Fos-positive cells in the TNC in the SUMA+MINO group, *n* = 4 per group. **(D,F)** Immunofluorescence staining of CGRP showed reduced immunofluorescence intensity in the TNC in the SUMA+MINO group, *n* = 4 per group (*p < 0.05, **p < 0.01, ***p < 0.001 and ****p < 0.0001; #*p* < 0.05, ^##^*p* < 0.01, ^###^*p* < 0.001 and ^####^*p* < 0.0001; *represents that the SUMA group was compared with the VEH group, ^#^represents that the SUMA group was compared with the SUMA+MINO group; scale bar = 50/200 μm).

Prior to the daily treatment of SUMA, a microglial inhibitor, minocycline (MINO), was injected intraperitoneally, which resulted in the inhibition of microglial activation. Iba1 and iNOS protein expression reduced dramatically in the SUMA+MINO group compared with the SUMA group (*p* < 0.01 and p < 0.05; Figures 3A–C), and the SUMA+MINO group also showed a significant decrease in iNOS mRNA levels (*p* < 0.05; Figure 3D). Similarly, a reduced number of Iba1 immunopositive cells was observed in the SUMA+MINO group, as shown by the immunofluorescence staining images (*p* < 0.0001, Figures 3E,F). The number of branches, average branch length and maximum branch length of microglia did not differ significantly between groups (Figure 3F, rightmost column). After microglial activation was inhibited, the reduction in mechanical pain threshold caused by repeated SUMA injection was ameliorated (*p* < 0.0001, Figures 4A,B), and c-Fos and CGRP expression was reduced in the TNC (*p* < 0.0001, Figures 4C–F). These findings suggest that inhibiting microglial activation can reduce central sensitization and hyperalgesia in MOH.

### 3.4. Increased expression of P2X7R in the TNC after repeated SUMA injection

**Figure 5:**
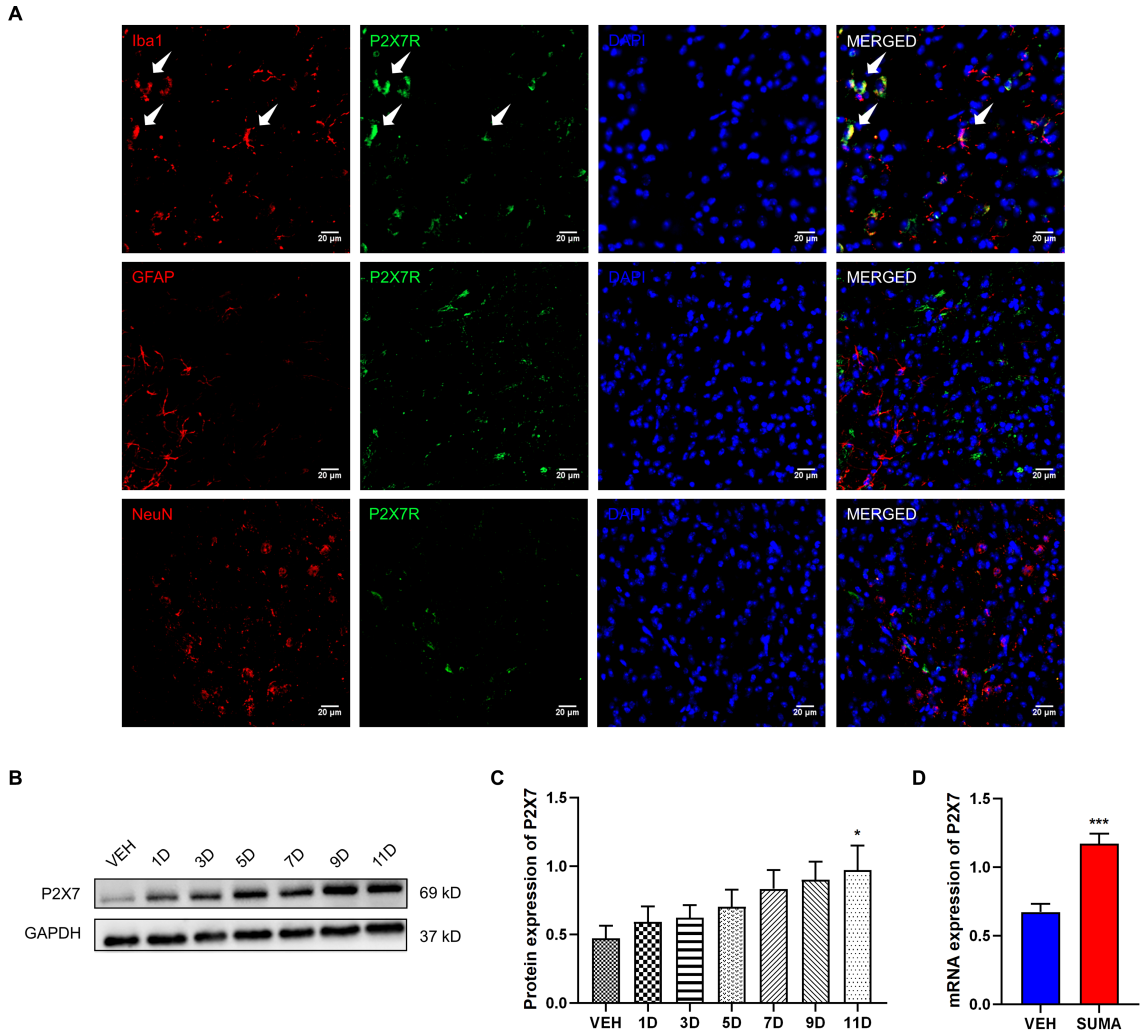
Increased P2X7R expression in the TNC after repeated SUMA injection **(A)** P2X7R is double immunofluorescently labeled in the TNC with Iba1, GFAP and NeuN. The white arrow indicates P2X7R colocalization mainly with Iba1. **(B,C)** The protein expression of P2X7R gradually grew with repeated SUMA injection, *n* = 6 per group. **(D)** Compared with the VEH group, the mRNA expression of P2X7R was greatly increased in the SUMA group, *n* = 6 per group (*p < 0.05, ***p < 0.001; *represents that the SUMA group was compared with the VEH group; scale bar = 20 μm).

Previous research has demonstrated that P2X7R is predominantly located in microglia (Jiang et al., 2021). Therefore, in this study, we further examined the function of microglial P2X7R in MOH mice. Double immunofluorescence labeling was used to examine the relationships between P2X7R and different cellular type-specific indicators in the TNC, including Iba-1 for microglia, GFAP for astrocytes and NeuN for neurons. Consistent with previous findings, our results showed that P2X7R colocalized primarily with Iba1 (shown by white arrows in Figure 5A) but not with NeuN or GFAP. The protein and mRNA expression levels of P2X7R were subsequently observed by western blotting and qRT–PCR. After repeated SUMA injection, P2X7R protein expression increased in a time-dependent way (*p* < 0.01, Figures 5B,C). Likewise, the mRNA expression in the SUMA group was much higher than that in the VEH group (*p* < 0.001, Figure 5D). Collectively, these findings demonstrated that repeated SUMA injection caused a progressively growth in microglial P2X7R in the TNC.

### 3.5. Repeated SUMA injection induced the activation of NLRP3 inflammasome in the TNC

**Figure 6:**
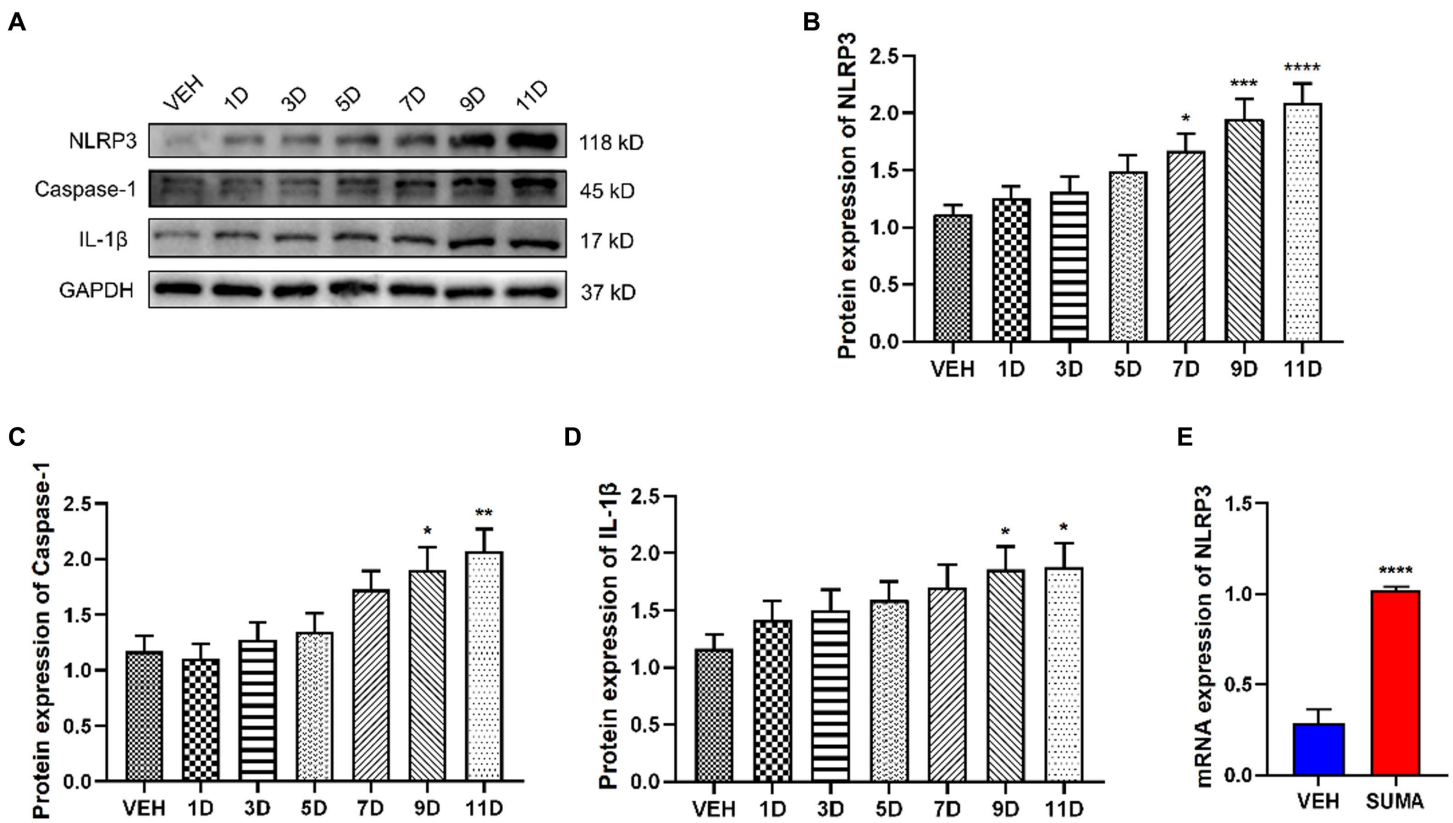
Repeated SUMA injection induced the NLRP3 inflammasome activation in the TNC. **(A–D)** NLRP3, Caspase-1 and IL-1β protein expression elevated gradually over time, *n* = 6 per group. **(E)** The mRNA expression of NLRP3 was progressively higher in the SUMA group than that in the VEH group, *n* = 6 per group (*p < 0.05, **p < 0.01, ***p < 0.001, and ****p < 0.0001, *represents that the SUMA group was compared with the VEH group).

It has been demonstrated in earlier studies that the NLRP3 inflammasome, which is downstream of P2X7R, is implicated in the pathophysiology of a variety of disorders (Territo and Zarrinmayeh, 2021; Li et al., 2022; Richter et al., 2022). Thus, we further investigated the changes in the NLRP3 inflammasome after repeated SUMA injection in this study. Our data demonstrated that following repeated SUMA injection, the protein expression of NLRP3, Caspase-1, and IL-1β elevated gradually over time in the TNC (*p* < 0.0001, *p* < 0.01, *p* < 0.05, respectively; Figures 6A–D), and the mRNA expression of NLRP3 in the MOH group raised dramatically compared with that in the VEH group (*p* < 0.0001, Figure 6E). These results suggested that repeated SUMA injections activated the NLRP3 inflammasome in the TNC.

### 3.6. Inhibiting P2X7R attenuated SUMA-induced hyperalgesia, the activation of NLRP3 inflammasome and the expression of c-Fos and CGRP in the TNC

**Figure 7:**
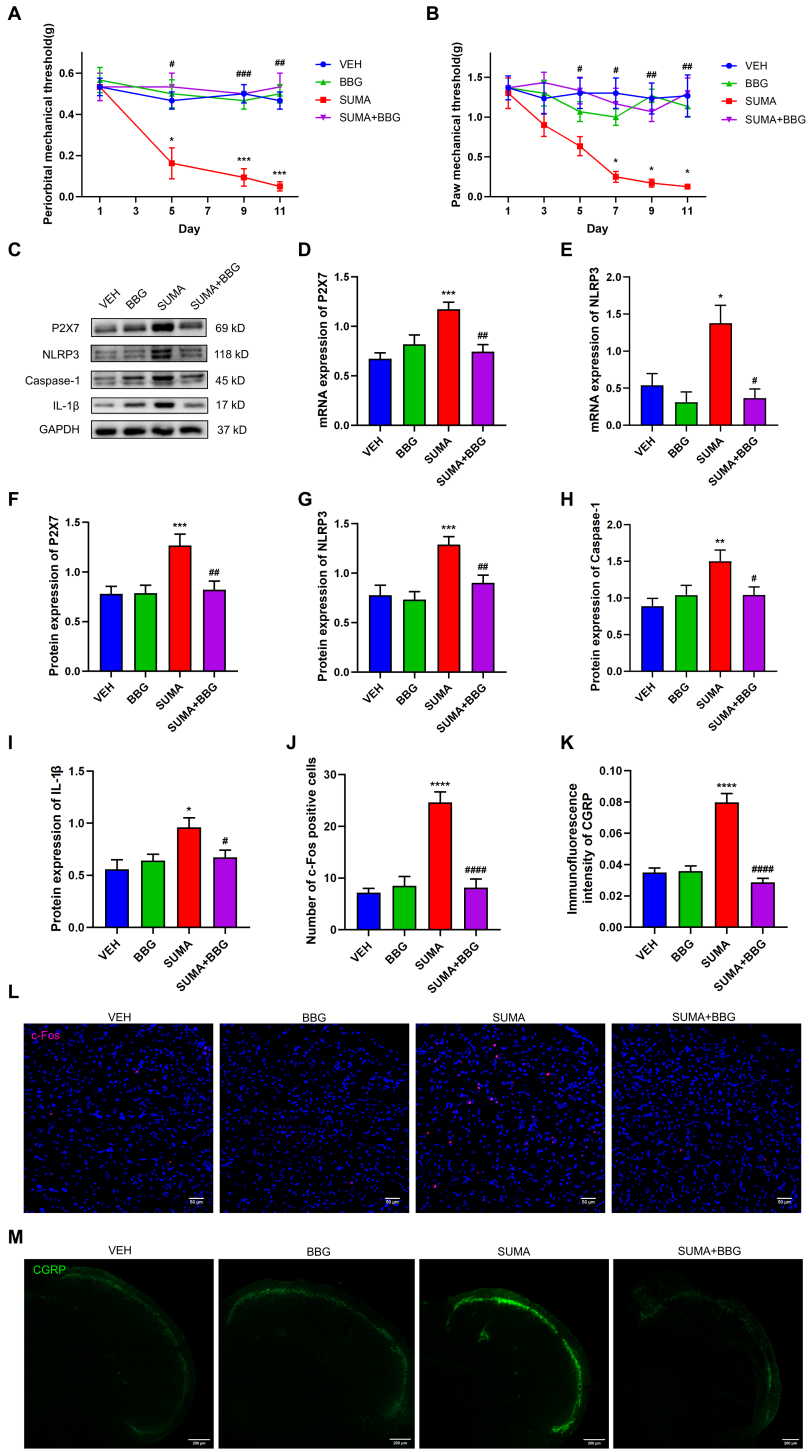
Inhibiting P2X7R attenuated SUMA-induced hyperalgesia, NLRP3 inflammasome activation and the of c-Fos and CGRP expression in the TNC. **(A,B)** The periorbital and hind paws mechanical withdrawal thresholds were significantly restored in the SUMA+BBG group, *n* = 6 per group. **(C,F–I)** The protein expression of P2X7R, NLRP3, Caspase-1 and IL1-β. *n* = 6 per group. **(D,E)** The mRNA expression of P2X7R and NLRP3, *n* = 6 per group. **(J,L)** Immunofluorescence staining of c-Fos in the TNC. c-Fos immunopositive cells were significantly reduced in the SUMA+BBG group compared with the SUMA group, *n* = 4 per group. **(K,M)** Immunofluorescence staining of CGRP in the TNC. The fluorescence intensity of CGRP was observably lower in the FIGURE 7 (Continued)SUMA+BBG group, *n* = 4 per group (*p < 0.05, **p < 0.01, ***p < 0.001 and ****p < 0.0001; ^#^*p* < 0.05, ^##^*p* < 0.01, ^###^*p* < 0.001, and ^####^*p* < 0.0001; *represents that the SUMA group was compared with the VEH group, ^#^represents that the SUMA group was compared with the SUMA + MINO group; scale bar = 50/200 μm).

To further determine the role of P2X7R, a P2X7R antagonist BBG was administered intraperitoneally to the mice before each SUMA injection. We discovered that BBG treatment greatly reduced mechanical hyperalgesia induced by SUMA in the periorbital and hind paws regions (Figures 7A,B). In comparison with the SUMA group, the SUMA+BBG group had lower P2X7R protein expression after BBG treatment (Figures 7C,F). Consistent with the decline in P2X7R, BBG treatment markedly reduced the protein expression of NLRP3, Caspase-1, and IL-1β (Figures 7C,G–I). In addition, the mRNA expression of P2X7R and NLRP3 was dramatically cut down (Figures 7D,E). It was also shown that the SUMA+BBG group and the SUMA group significantly differed in the expression of c-Fos and CGRP (Figures 7J–M). Based on these observations, inhibiting P2X7R could prevent hyperalgesia and central sensitization in MOH mice, and this effect was mediated by a reduction in NLRP3 inflammasome activation.

### 3.7. Inhibiting the NLRP3 inflammasome attenuated SUMA-induced hyperalgesia and c-Fos and CGRP expression in the TNC

**Figure 8:**
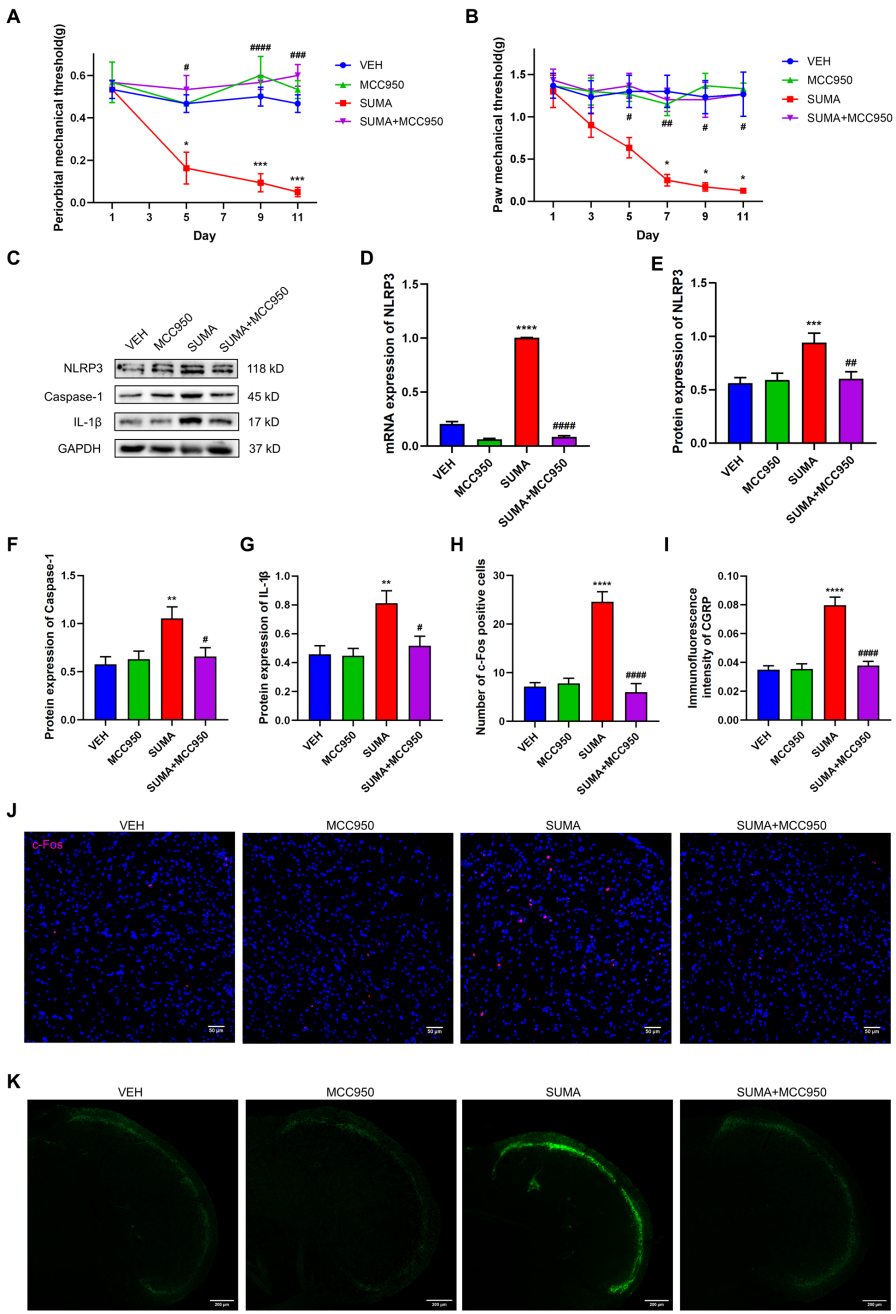
Inhibiting the NLRP3 inflammasome attenuated SUMA-induced hyperalgesia and the expression of c-Fos and CGRP in the TNC. **(A,B)** The periorbital and hind paw mechanical withdrawal thresholds were markedly restored in the SUMA+ MCC950 group in comparison with the SUMA group, *n* = 6 per group. **(C,E–G)** The protein expression of NLRP3, Caspase-1 and IL-1β. *n* = 6 per group. **(D)** The NLRP3 mRNA expression levels. *n* = 6 per group. **(H,J)** Immunofluorescence staining of c-Fos in the TNC. When compared with the SUMA group, the number of c-Fos-positive cells was considerably lower in the SUMA+ MCC950 group, *n* = 4 per group. **(I,K)** Immunofluorescence staining of CGRP in the TNC. The immunofluorescence intensity of CGRP was significantly lower in the SUMA + MCC950 group, *n* = 4 per group (*p < 0.05, **p < 0.01, ***p < 0.001, and ****p < 0.0001; ^#^*p* < 0.05, ^##^*p* < 0.01, ^###^*p* < 0.001, and ^####^p < 0.0001; *represents that the SUMA group was compared with the VEH group, ^#^represents that the SUMA group was compared with the SUMA+MINO group; scale bar = 50/200 μm).

To inhibit the activity of the NLRP3 inflammasome, the NLRP3-specific inhibitor MCC950 was injected intraperitoneally prior to the daily administration of SUMA. The mechanical hyperalgesia caused by SUMA in the periorbital and hind paws was significantly suppressed by MCC950 (*p* < 0.05; Figures 8A,B). The protein levels of NLRP3, caspase-1, and IL-1β, as well as the mRNA levels of NLRP3, were markedly decreased by MCC950 injection (*p* < 0.001; Figures 8C–G). Moreover, MCC950 significantly restored the expression of c-Fos and CGRP in the TNC induced by repeated SUMA injection (*p* < 0.0001; Figures 8H–K). These findings revealed that NLRP3 inhibition could reduce hyperalgesia and central sensitization in MOH mice.

## 4. Discussion

We presented several novel findings in the current study. First, we observed the activation of microglia in the TNC in a SUMA-induced MOH model and determined that inhibiting microglial activation mitigated hyperalgesia in MOH mice. Following that, we found that the microglial P2X7R/NLRP3 inflammasome in the TNC was activated and engaged in the pathophysiology of MOH. Our data indicate that microglial P2X7R regulates the activation of the NLRP3 inflammasome, which might be an underlying mechanism of MOH.

We employed a reported MOH animal model that was created by repeatedly giving the rodents SUMA, an acute analgesic for migraineurs (Rau et al., 2020; Ikegami et al., 2022). Previous studies have shown that male and female mice can show a decrease in periorbital and hind paws withdrawal thresholds as an answer to mechanical stimulation after repeated exposure to SUMA (Moye et al., 2019; Ikegami et al., 2022). In this study, it was observed that repeated SUMA injection induced hyperalgesia in the periorbital and hind paws of mice, and there was no gender difference. This hyperalgesia was time dependent, and the degree of hyperalgesia progressively worsened with time. Our findings are in line with prior research on the MOH model, but to avoid potential sex differences (Levine et al., 2020; Ikegami et al., 2022), only male mice were studied in follow-up studies.

Although the exact mechanism of MOH is unknown, studies have revealed that the development of central sensitization may be a critical factor in MOH. Overuse of paracetamol enhanced the incidence of cortical spreading depression (CSD) and the quantity of CSD-induced c-Fos immunoreactive positive cells in the TNC in rats (Supornsilpchai et al., 2010a,b). Increased expression of CGRP or substance P was observed in the TNC and TG after repeated exposure to triptans (de Felice et al., 2010a,b; Urru et al., 2022). Although MOH does not present exclusively as migraine, in most patients with triptan-associated MOH, the headache form is similar to migraine (Limmroth et al., 2002). Numerous previous studies have demonstrated that central sensitization is one of the most significant pathophysiological processes of chronic migraine (Long et al., 2018; Wen et al., 2021). In the chronic migraine animal model, prolonged excitability of trigeminal-cervical complex (TCC) neurons and incremental expression of c-Fos and CGRP within the TNC were observed (Woolf, 2011; Su and Yu, 2018; Pan et al., 2022). Thus, in this research, we assessed the state of central sensitization after repeated SUMA injection by measuring the expression of c-Fos and CGRP. We observed increased levels of c-Fos and CGRP in the TNC after repeated SUMA administration, which was consistent with earlier results.

Microglia is a special type of macrophages located in the CNS. There is now ample evidence that these cells play a crucial role in central sensitization. Numerous inflammatory substances, including nitric oxide, TNF-α, and IL-1β, are generated and released by activated microglia, which can directly and indirectly increase neuronal excitability and induce central sensitization (Johnson et al., 2013). In addition to the effects on neurons, these inflammatory substances can further excite microglia, creating a positive feedback loop that in turn exacerbates central sensitization. Studies have demonstrated that long-term morphine exposure stimulates astrocytes and microglia in the spinal cord (Mika, 2008). However, a subsequent study showed that continuous intrathecal morphine injection significantly increased the quantity of p-p38-positive cells in the spinal cord. p-p38 colocalized primarily with activated spinal microglia but not astrocytes (Cui et al., 2006). Similarly, animal studies of chronic migraine revealed microglial activation in the TNC, and inhibiting microglial activation improved the central sensitization state in chronic migraine animals (Long et al., 2018). Accordingly, the function of microglia in MOH was the main topic of this study. Our study showed that after repeated SUMA injection, the microglia in the TNC were activated, mainly manifested as an increase in the protein expression, mRNA expression and number of microglia (Schwabenland et al., 2021). The mechanical hyperalgesia induced by repeated SUMA injection was alleviated, and the expression of c-Fos and CGRP in the TNC was reduced after the administration of the microglia inhibitor minocycline. Microglia may be involved in the MOH animal model established by repeated SUMA injection.

Likewise, P2X7R plays an important pathophysiological role in chronic pain, including migraine, morphine analgesic tolerance, and neuropathic pain. It was found that after repeated exposure to opioids such as morphine, animals developed mechanical hyperalgesia and had upregulated P2X7R expression in the gray matter sites of the midbrain conduits and spinal cord. The use of P2X7R inhibitors inhibited the reduction in mechanical withdrawal thresholds (Zhou et al., 2010; Xiao et al., 2015). An earlier research of our team demonstrated that microglial P2X7R contributes to the onset of chronic migraine through controlling autophagy (Jiang et al., 2021). Extensive studies have shown that among the multiple signaling pathways activated by P2X7R, reduced intracellular K^+^, increased intracellular Ca^2+^ and mitochondrial depolarization can further lead to NLRP3 inflammasome activation (Franceschini et al., 2015; Swanson et al., 2019). Pro-caspase-1 is cleaved by the NLRP3 inflammasome to create mature caspase-1, and then pro-IL-1β is cleaved to create mature IL-1β, which is involved in neuroinflammation. Our prior research showed that the NLRP3 inflammasome was stimulated in an animal model of chronic migraine (He et al., 2019). Consistent with earlier studies (Zhang et al., 2022), our findings demonstrated that P2X7R was primarily located in microglia in the TNC and that the upregulation of P2X7R and the NLRP3 inflammasome induced by repeated SUMA injection paralleled MOH-associated mechanical hyperalgesia. We determined the effects of the P2X7R inhibitor BBG and the NLRP3 inhibitor MCC950 on MOH mice in order to further confirm the function of P2X7R and NLRP3 inflammasome activation in MOH. Daily BBG injection not only significantly inhibited mechanical hyperalgesia induced by repeated SUMA injection but also suppressed NLRP3 inflammasome activation and reduced the expression of c-Fos and CGRP. Mechanical hyperalgesia and elevated c-Fos and CGRP expression were similarly inhibited by daily injection of MCC950.

Thus, our results indicate that microglia in the TNC are engaged in pathophysiological processes in an animal model of MOH via the P2X7R/NLRP3 inflammasome. To date, no studies have examined the function and specific mechanisms of microglia in MOH. Our current study fills this gap.

However, there are shortcomings in this study. First, only male mice were used for subsequent studies after we observed no significant differences in behavior data between male and female mice. However, previous pain-related study has shown that there are differences in the role of microglia in mice of different genders (Sorge et al., 2015). This may become a key direction in the study of the mechanism of MOH. Second, various inhibitors were systematically used, and we cannot exclude the possibility that there may be potential effects on other pain-modulating regions. Finally, in previous MOH-related animal studies, hyperalgesia returned after the administration of SUMA was stopped, which is in line with the clinical observation that headaches can improve in MOH patients after drug cessation. However, changes after the cessation of SUMA injection were not observed in this study, and whether the molecular mechanism is related to microglia needs further investigation.

## 5. Conclusion

Our data suggest that hyperalgesia induced by repeated SUMA injection can activate microglia in the TNC. Microglia mediate central sensitization by promoting activation of the P2X7R/NLRP3 inflammasome, which elevates IL-1β expression and thereby participates in pathophysiological processes in animal models of MOH. Inhibiting microglia or the P2X7R/NLRP3 inflammasome attenuated mechanical hyperalgesia and central sensitization in MOH mice. Consequently, the microglial P2X7R/NLRP3 inflammasome may be a prospective therapeutic target for treatment of MOH resulting from the repeated use of acute analgesics for migraine.

## Data availability statement

The original contributions presented in the study are included in the article/supplementary material, further inquiries can be directed to the corresponding author.

## Ethics statement

The animal study was reviewed and approved by The Animal Care and Use Committee of Chongqing Medical University.

## Author contributions

YW and LD designed all experiments, performed the experiments, and wrote the manuscript. WH participated in data analysis. YuZ, YiZ, GQ, and DZ provided assistance and support for experimental design and fluorescence manipulation. LC, WH, and JZ provided financial support and revised this manuscript. All authors contributed to the article and approved the submitted version.

## Funding

Funding support provided by the National Natural Science Foundation of China (No: 81971063).

## Conflict of interest

The authors declare that the research was conducted in the absence of any commercial or financial relationships that could be construed as a potential conflict of interest.

## Publisher’s note

All claims expressed in this article are solely those of the authors and do not necessarily represent those of their affiliated organizations, or those of the publisher, the editors and the reviewers. Any product that may be evaluated in this article, or claim that may be made by its manufacturer, is not guaranteed or endorsed by the publisher.

## Glossary

**Table:**

MOH	medication overuse headache
ICHD-3	International Classification of Headache Disorders, Third Edition
TNC	trigeminal nucleus caudalis
SUMA	sumatriptan
CGRP	calcitonin gene-related peptide
Iba1	Ionized calcium-binding adaptor molecule-1
iNOS	Inducible nitric oxide synthase
P2X7R	P2X7 receptor
BBG	Brilliant Blue G
NLRP3	nucleotide-binding oligomeric domain-like receptor protein 3
NSAIDs	nonsteroidal anti-inflammatory drugs
CNS	central nervous system
ASC	apoptosis-associated speck-like protein containing CARD
Caspase-1	cysteine-aspartate protease-1
IL-1β	Interleukin-1β
NTG	nitroglycerin
NIH	National Institutes of Health
qRT-PCR	Quantitative real-time polymerase chain reaction
GAPDH	glyceraldehyde-3-phosphate dehydrogenase
PMSF	phenylmethylsulfonyl fluoride
SDS–PAGE	sodium dodecyl sulfate polyacrylamide
PVDF	polyvinylidene difluoride
TBST	Tris-buffered saline with Tween-20
PBS	phosphate-buffered saline
PFA	paraformaldehyde
DAPI	4′,6-diamidino-2-phenylindole
SEM	standard error
ANOVA	One-way analysis of variance
MINO	minocycline
CSD	cortical spreading suppression
TCC	trigeminal-cervical complex
TNF-α	tumor necrosis factor-α
